# Update on Shoulder Arthroplasties with Emphasis on Imaging

**DOI:** 10.3390/jcm12082946

**Published:** 2023-04-18

**Authors:** Jennifer S. Weaver, Imran M. Omar, Nicholson S. Chadwick, Joanna L. Shechtel, Jamie M. Elifritz, Christopher L. Shultz, Mihra S. Taljanovic

**Affiliations:** 1 Department of Radiology and Radiologic Sciences, Vanderbilt University Medical Center, 1161 21st Ave. S, MCN CCC-1118, Nashville, TN 37232, USA; 2 Department of Radiology, Northwestern Memorial Hospital, 676 N. Saint Clair Street, Suite 800, Chicago, IL 60611, USA; 3 Department of Radiology, MSC08 4720, 1 University of New Mexico, Albuquerque, NM 87131, USA; 4 Department of Pathology, University of New Mexico, New Mexico Office of the Medical Investigator, MSC08 4720, 1 University of New Mexico, Albuquerque, NM 87131, USA; 5 Department of Orthopaedics and Rehabilitation, University of New Mexico, MSC 10 5600, 1 University of New Mexico, Albuquerque, NM 87131, USA; 6 Department of Medical Imaging, University of Arizona, 1501 N. Campbell, Tucson, AZ 85724, USA

**Keywords:** shoulder, rotator cuff, arthritis, arthroplasty, radiography, computed tomography, magnetic resonance imaging, ultrasound

## Abstract

Shoulder pain and dysfunction may significantly impact quality of life. If conservative measures fail, advanced disease is frequently treated with shoulder arthroplasty, which is currently the third most common joint replacement surgery following the hip and knee. The main indications for shoulder arthroplasty include primary osteoarthritis, post-traumatic arthritis, inflammatory arthritis, osteonecrosis, proximal humeral fracture sequelae, severely dislocated proximal humeral fractures, and advanced rotator cuff disease. Several types of anatomic arthroplasties are available, such as humeral head resurfacing and hemiarthroplasties, as well as total anatomic arthroplasties. Reverse total shoulder arthroplasties, which reverse the normal ball-and-socket geometry of the shoulder, are also available. Each of these arthroplasty types has specific indications and unique complications in addition to general hardware-related or surgery-related complications. Imaging—including radiography, ultrasonography, computed tomography, magnetic resonance imaging, and, occasionally, nuclear medicine imaging—has a key role in the initial pre-operative evaluation for shoulder arthroplasty, as well as in post-surgical follow-up. This review paper aims to discuss important pre-operative imaging considerations, including rotator cuff evaluation, glenoid morphology, and glenoid version, as well as to review post-operative imaging of the various types of shoulder arthroplasties, to include normal post-operative appearances as well as imaging findings of complications.

## 1. Introduction

**Figure 1:**
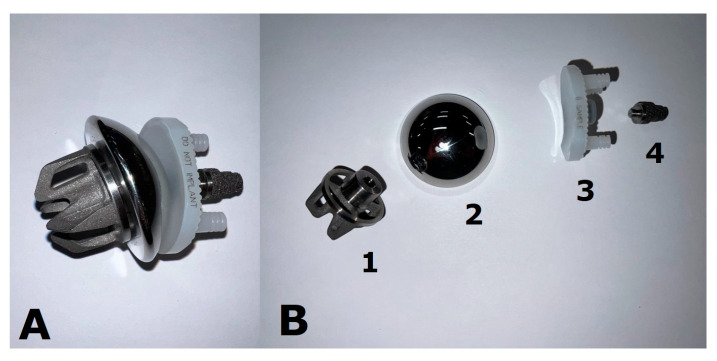
Example of a stemless shoulder implant. This prosthesis is shown (**A**) assembled and (**B**) deconstructed. The humeral head is secured to the cut humeral surface via an “anchor”, which is fluted and ridged to facilitate osseous integration. The anchor (1) and humeral head (2) are joined via a Morse taper. This implant allows for the preservation of humeral bone stock and functions as a resurfacing-type construct. The glenoid component (3) is composed of polyethylene plastic. The top and bottom pegs are cemented in place, while the metal post (4) is press-fit and allows for biologic osseous integration.

**Figure 2:**
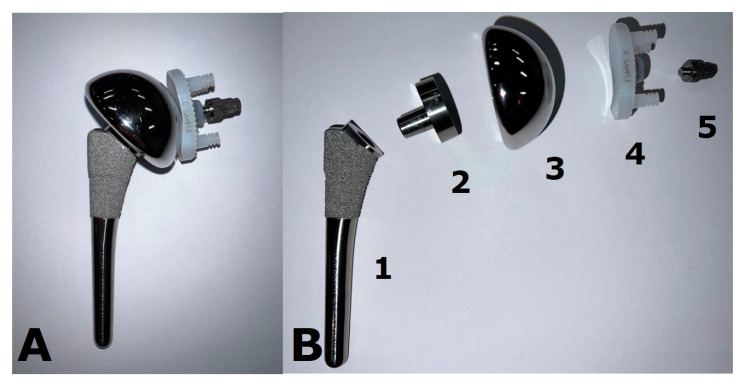
Example of an anatomic total shoulder arthroplasty (ATSA) implant. This prosthesis is shown (**A**) assembled and (**B**) deconstructed. The stem (1) is porous-coated proximally to allow for biologic osseous integration. The humeral head (3) is fixed to the stem via a Morse-tapered trunnion (2). Note the asymmetry of the trunnion, which allows for matching of the eccentricity of the native humeral head. The glenoid component (4) is composed of polyethylene plastic. The top and bottom pegs are cemented in place, while the metal post (5) is press-fit and allows for biologic osseous integration.

**Figure 3:**
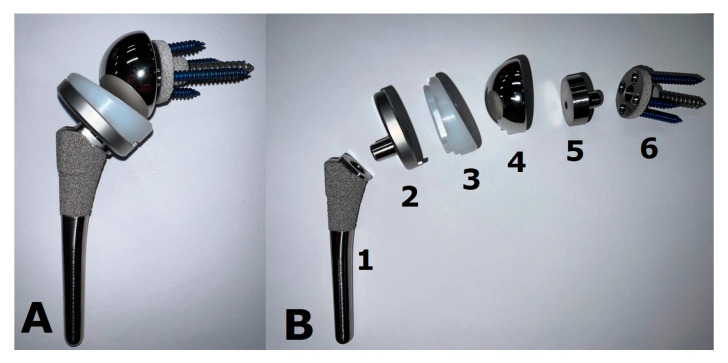
Example of a reverse total shoulder arthroplasty (RTSA) implant. This prosthesis is shown (**A**) assembled and (**B**) deconstructed. The stem (1) is porous-coated proximally to allow for biologic osseous integration. The humeral tray (2) is fixed to the stem via a Morse-tapered trunnion and articulates via a polyethylene liner (3). The glenoid base plate/metaglene (6) is porous-coated as well to facilitate osseous integration. It is fixed via a central screw that provides compression and is reinforced with locking screws peripherally. The glenosphere (4) attaches to the glenoid baseplate/metaglene via an eccentric Morse taper (5) to allow for distalization of the components and minimize scapular notching.

Shoulder pain is a very common condition and can be debilitating. Several conservative therapies exist, such as exercise, physical therapy, and non-steroidal anti-inflammatory drugs. Additionally, ultrasound-guided procedures such as joint or bursal corticosteroid injections, fluid aspirations, and nerve blocks can be used [1]. Shoulder arthroplasty has greatly increased in popularity over the recent years and is currently the third most common joint-replacement surgery following hip and knee arthroplasty [2]. It can be used to treat a variety of conditions, including primary osteoarthritis (OA), post-traumatic arthritis, inflammatory arthritis, osteonecrosis, certain fractures, and advanced rotator cuff disease, with the goal of alleviating pain and restoring function. The use of shoulder arthroplasty following appropriately treated post-infectious arthritis remains a debated topic. Several types of implants are available, such as humeral head resurfacing arthroplasties (HHRA), hemiarthroplasties (HA), stemless arthroplasties (Figure 1), anatomic total shoulder arthroplasties (ATSA) (Figure 2), and reverse total shoulder arthroplasties (RTSA) (Figure 3). Each arthroplasty type has specific biomechanical features, with particular indications and unique complications in addition to general hardware-related or surgery-related complications.

**Figure 4:**
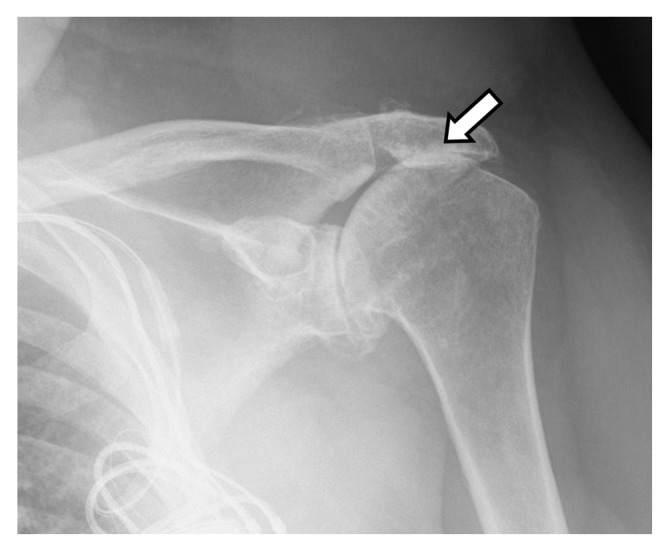
Frontal radiograph shows superior migration (white arrow) of the humeral head and developing acetabularization of the acromion in a patient with a full thickness rotator cuff tear.

**Figure 5:**
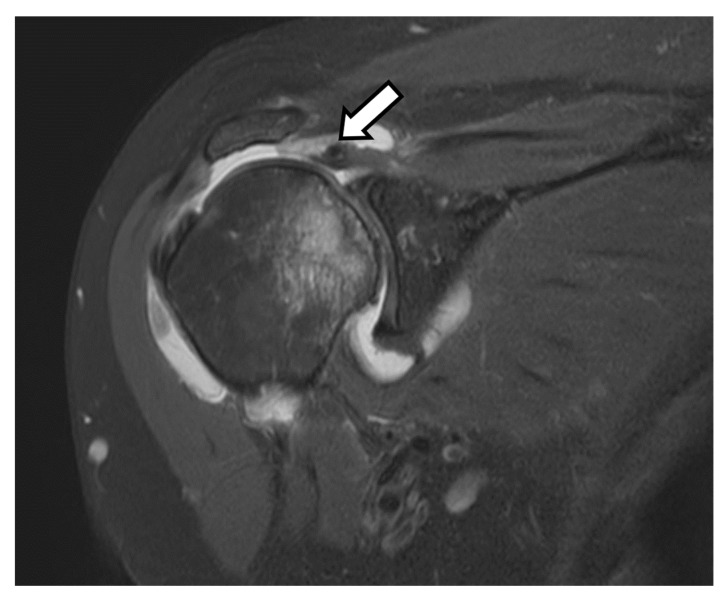
Coronal T2-weighted fat-saturated magnetic resonance (MR) image shows full thickness superior cuff tear with tendon retraction close to the level of the glenoid (arrow). Bone marrow edema and developing cartilage loss along the humeral head is consistent with developing cuff tear arthritis.

**Figure 6:**
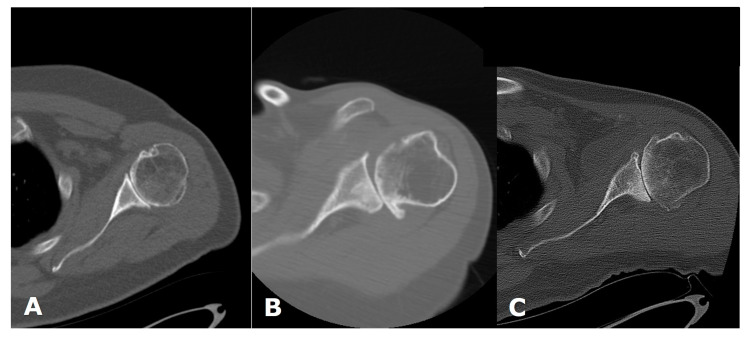
Axial computed tomography (CT) images of the shoulder in three different patients, showing different glenoid morphologies. (**A**). Walch A. (**B**). Walch B1. (**C**). Walch B2.

**Figure 7:**
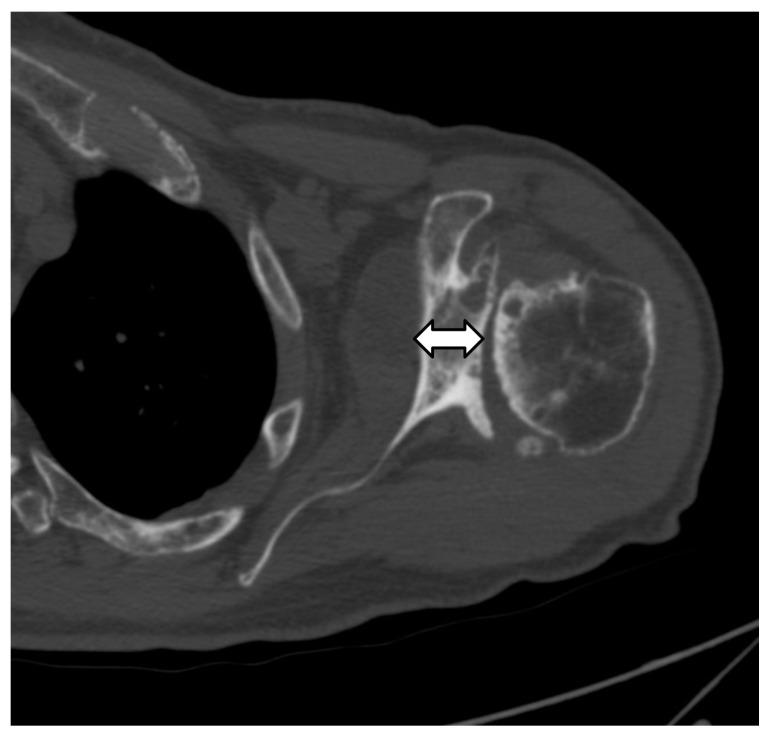
Axial CT image shows decreased glenoid bone stock (double arrow) due to glenoid erosion and retroversion.

**Figure 8:**
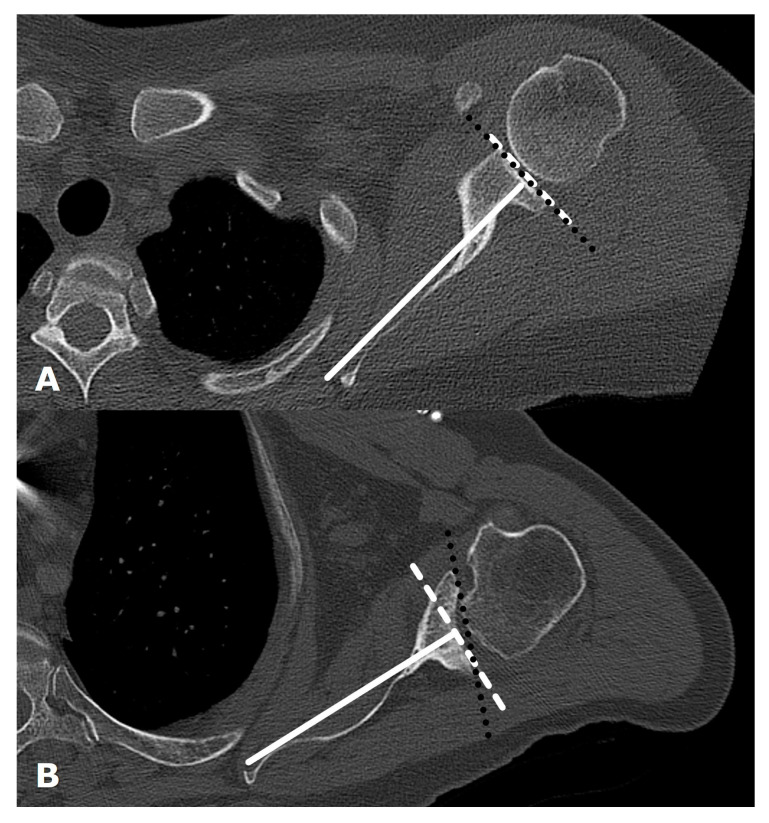
Axial CT images from two different patients (**A**). Normal glenoid version. (**B**). Retroversion secondary to glenoid erosion from osteoarthritis. A line (solid white) is drawn from the superior medial scapular border to the center of the glenoid and a line (white dashed) is drawn perpendicular to this on the axial image at or just inferior to the tip of the coracoid. Glenoid version is the angle formed between the perpendicular line (white dashed) and a line (black dotted) drawn along the glenoid articular surface.

**Figure 9:**
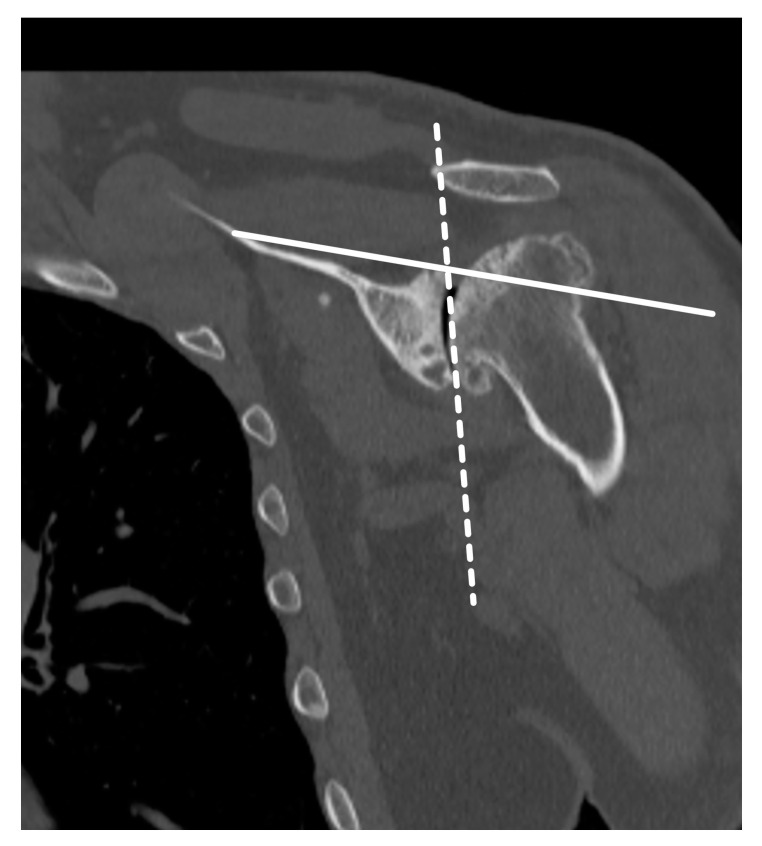
Coronal CT image showing glenoid inclination measurement. The inclination angle is measured between a line drawn along the supraspinatus fossa (solid line) and a second line along the glenoid fossa (dotted line).

**Figure 10:**
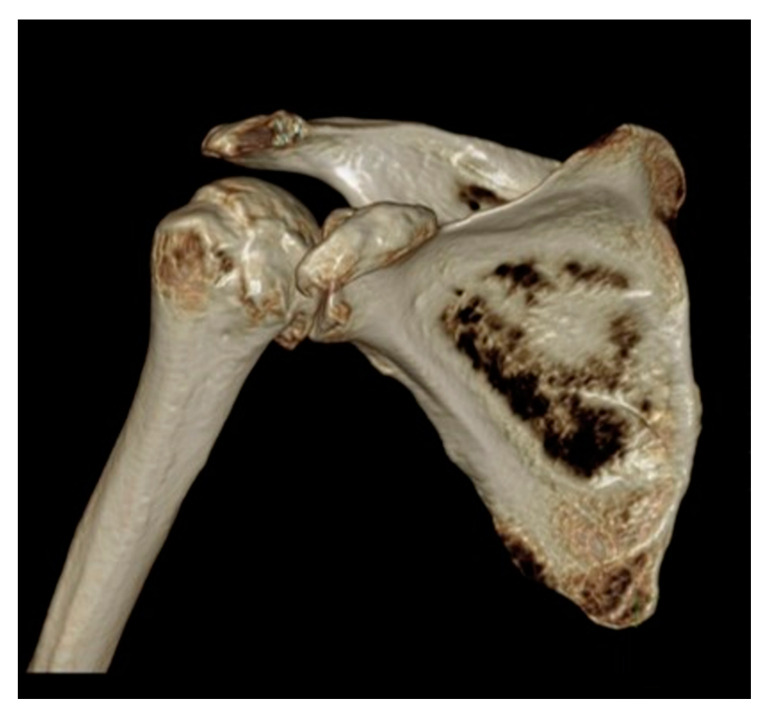
Three-dimensional reconstructed CT image of the shoulder in a patient with glenohumeral osteoarthritis, for preoperative planning.

**Figure 11:**
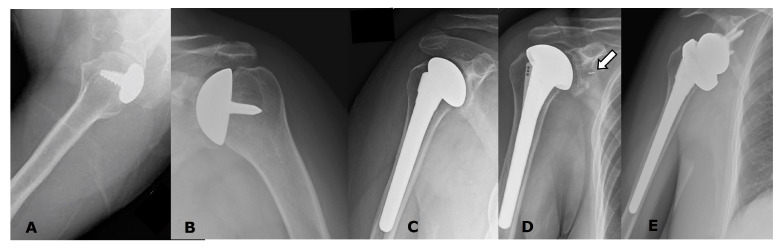
Radiographs of several different uncomplicated arthroplasty types. (**A**). Partial humeral head resurfacing arthroplasty (HHRA). (**B**). Humeral head resurfacing arthroplasty (HHRA). (**C**). Hemiarthroplasty (HA). (**D**). ATSA. Note small radiopaque marker identifying the glenoid component (arrow). (**E**). RTSA.

**Figure 12:**
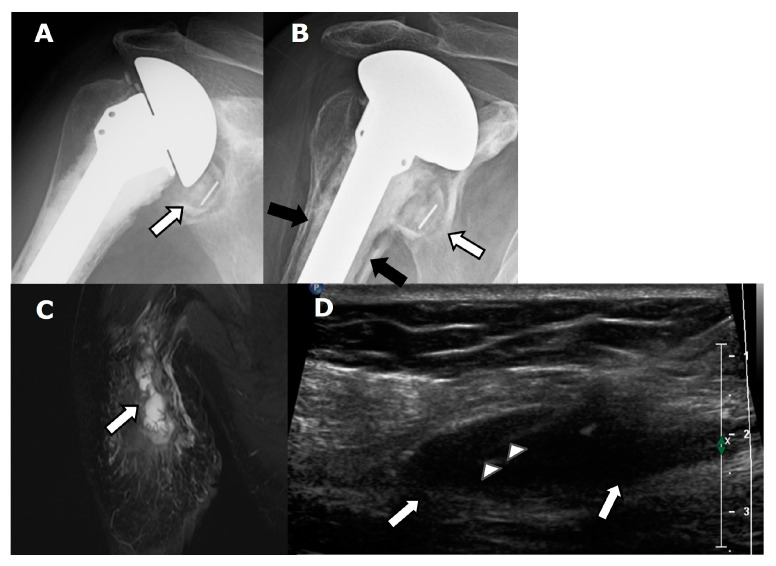
Patient with ATSA and periprosthetic joint infection in a patient with shoulder pain. (**A**). Frontal radiograph shows an ATSA with a small amount of lucency (arrow) around the glenoid component. (**B**). Frontal radiograph obtained several months later shows worsening lucency (white arrow) along the glenoid component, with new lucency (black arrows) along the bone–cement interface of the humeral component. (**C**). Coronal T2-weighted, fat-saturated MR image shows lobulated high signal (arrow) throughout the shoulder musculature. (**D**). Long-axis ultrasound (US) image shows an ovoid soft tissue fluid collection/abscess (arrows), with needle (arrowheads) present during diagnostic aspiration.

**Figure 13:**
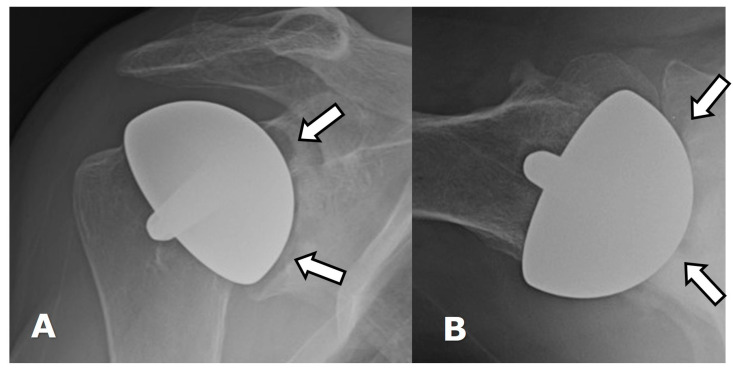
(**A**) Frontal and (**B**) axillary radiographs show glenoid wear with erosions (arrows) in a patient with an HHRA.

**Figure 14:**
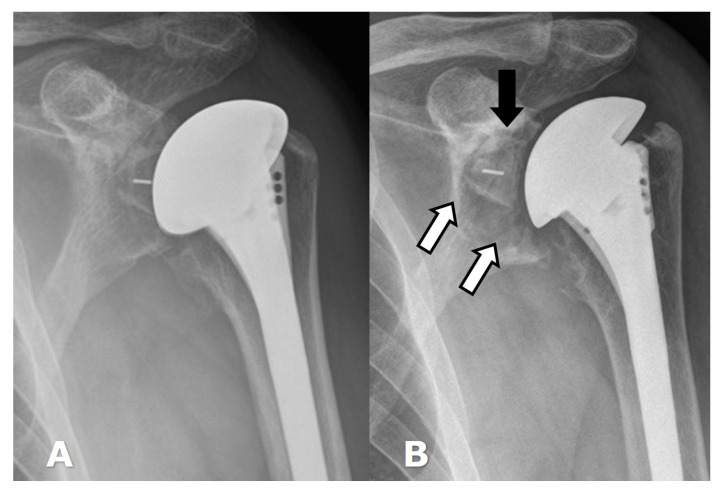
(**A**). Frontal radiograph in a patient with a normal-appearing ATSA. (**B**). Frontal radiograph several years later when patient presented with shoulder pain, showing lucency (white arrows) along the glenoid component, with slight superior rotation (black arrow) of the glenoid component within the bone, consistent with glenoid component loosening.

**Figure 15:**
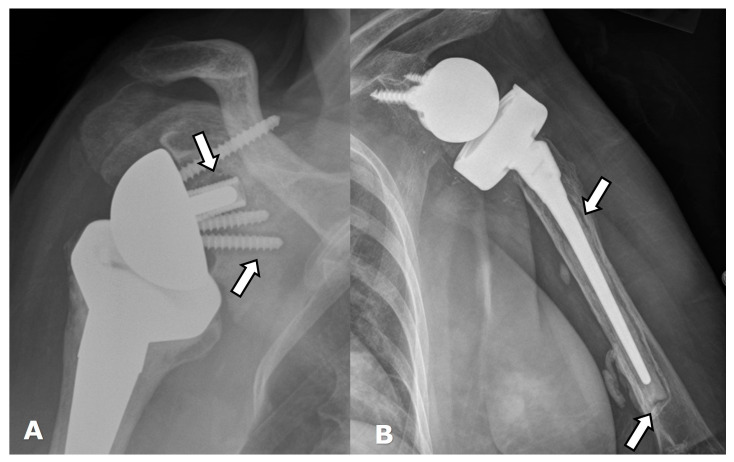
(**A**) Frontal radiograph in a patient with an RTSA shows lucency (arrows) along the baseplate screws and central screw consistent with loosening. (**B**). Frontal radiograph in a different patient with RTSA shows lucency (arrows) around the humeral component as well as the bone–cement interface, consistent with loosening. Cortical thinning is also noted along the medial aspect of the distal humeral stem.

**Figure 16:**
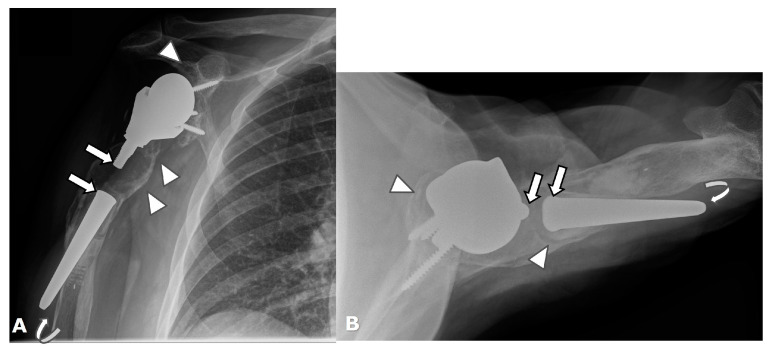
(**A**) Frontal and (**B**) axillary radiographs in a patient with RTSA showing failure of the humeral component, now separated into two separate pieces (white arrows). There is significant lucency (arrowheads) along the humeral and glenoid components, with cortical thinning, suggesting loosening or infection. The distal tip of the humeral component (curved arrow) breached the cortex and became extraosseous in location.

**Figure 17:**
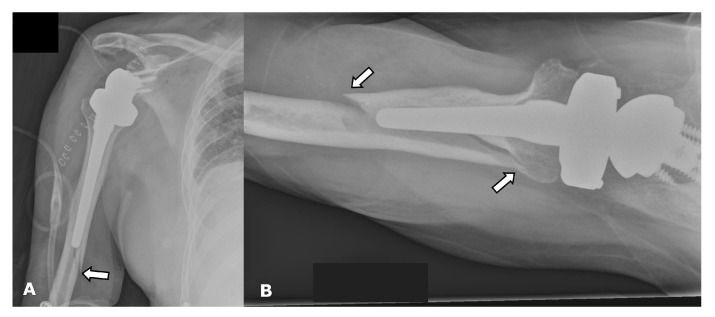
(**A**). Frontal radiograph in a patient immediately post-operative from RTSA shows a periprosthetic humeral fracture (arrow). (**B**). Axillary radiograph in a different patient with RTSA shows a periprosthetic fracture along the humeral stem (arrows).

**Figure 18:**
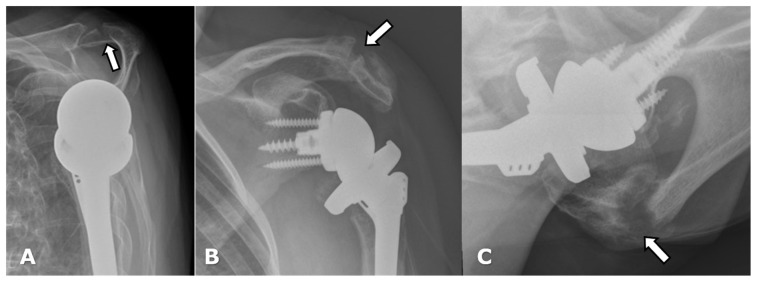
(**A**) Scapular Y radiograph in a patient with an RTSA shows an acute acromial fracture (arrow). (**B**) Frontal and (**C**) axillary radiographs in a different patient demostrate a healing acromial fracture (arrow) following RTSA.

**Figure 19:**
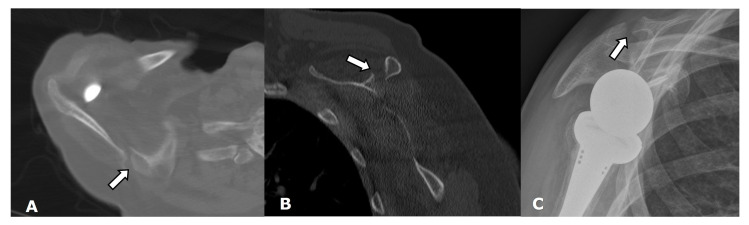
(**A**) Axial and (**B**) parasagittal CT images and (**C**) a scapular Y radiograph demonstrate a scapular fracture (arrow) following RTSA.

**Figure 20:**
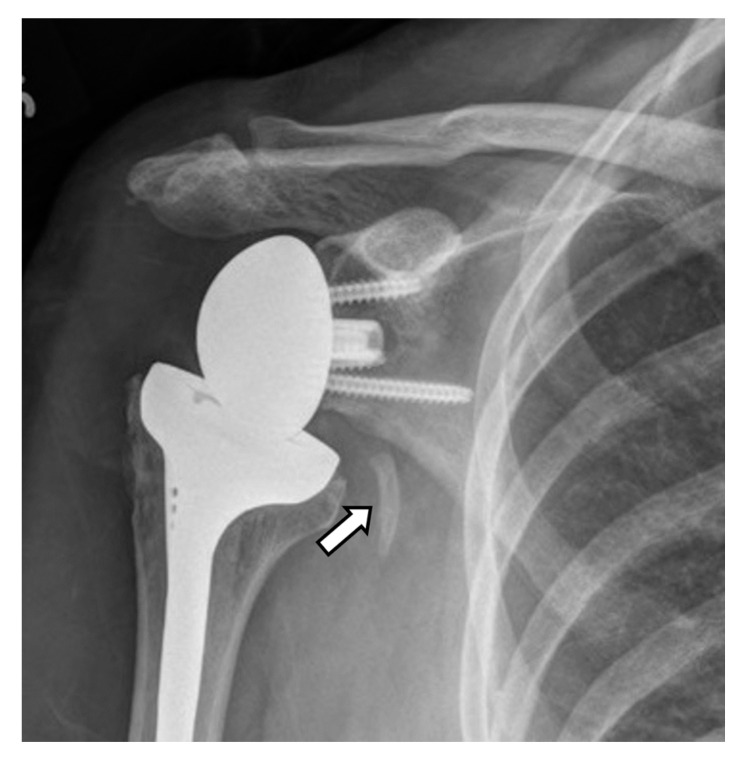
Frontal radiograph shows heterotopic ossification (arrow) along the proximal medial aspect of the humeral stem of an RTSA.

**Figure 21:**
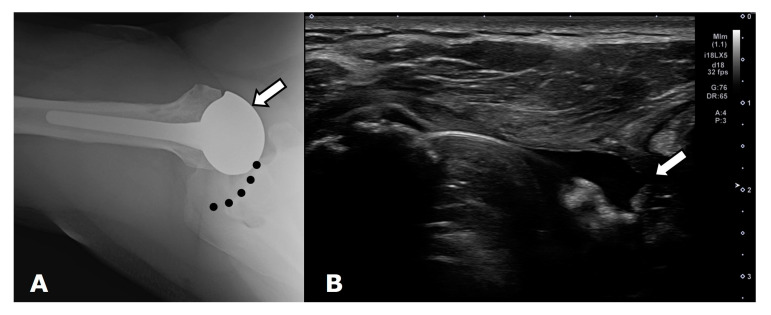
(**A**). Axillary radiograph shows anterior subluxation of the humeral head (arrow) component of the HA from the glenoid (dotted line) in a patient with subscapularis insufficiency. (**B**). Long-axis US image (lateral image left, medial image right) in a different patient with an ATSA shows a full thickness subscapularis tendon tear with a retracted tendon stump (arrow).

**Figure 22:**
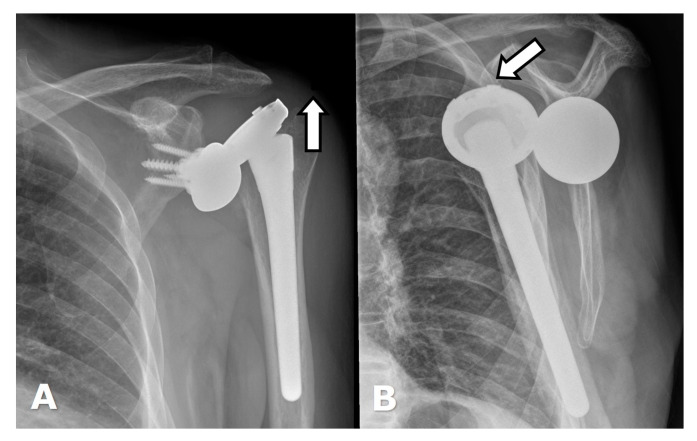
Dislocated RTSA. (**A**). Frontal radiograph shows the humeral component displaced superiorly (arrow denotes force direction). (**B**). Scapular Y view shows the humeral component displaced anteriorly (arrow denotes force direction).

**Figure 23:**
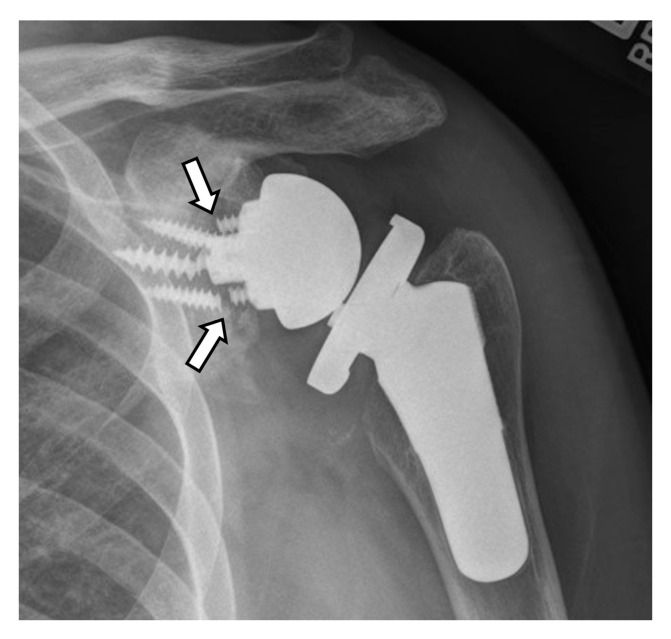
Frontal radiograph in a patient with an RTSA shows fracture/break (arrows) of three of the baseplate screws.

**Figure 24:**
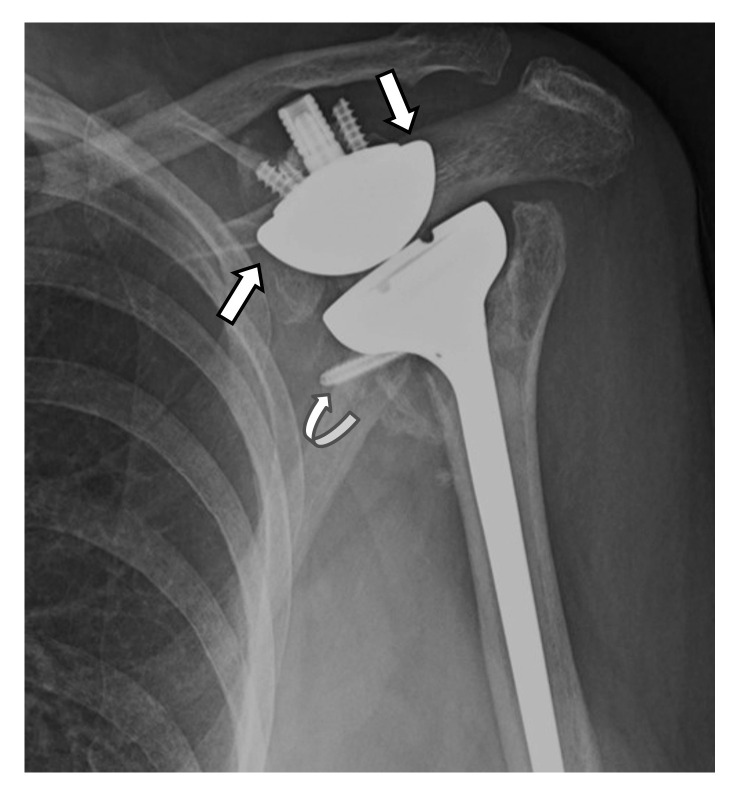
Frontal radiograph in a patient with RTSA shows fracture/break of a screw (curved arrow) as well as superior migration and rotation of the baseplate/metaglene (arrows), now dissociated from the glenoid.

**Figure 25:**
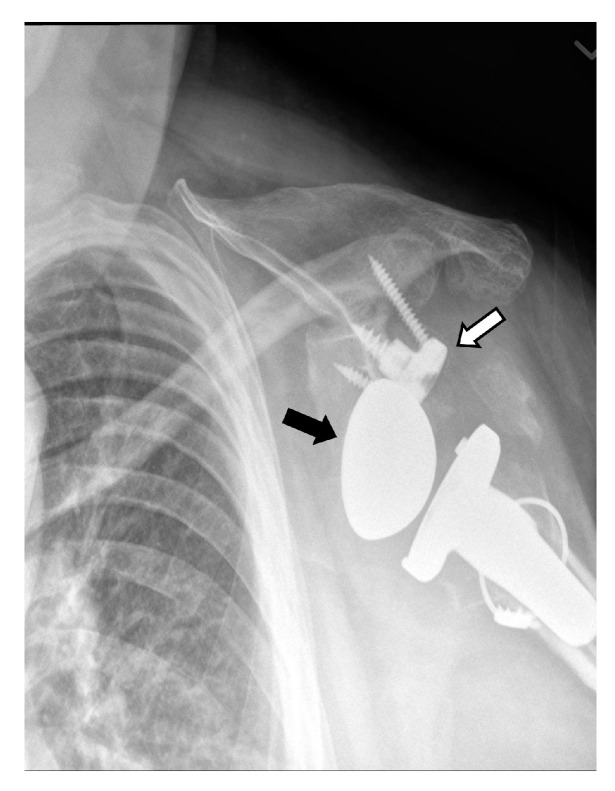
Frontal radiograph in a patient with RTSA shows dissociation of the metaglene/baseplate (white arrow) and the glenosphere (black arrow).

**Figure 26:**
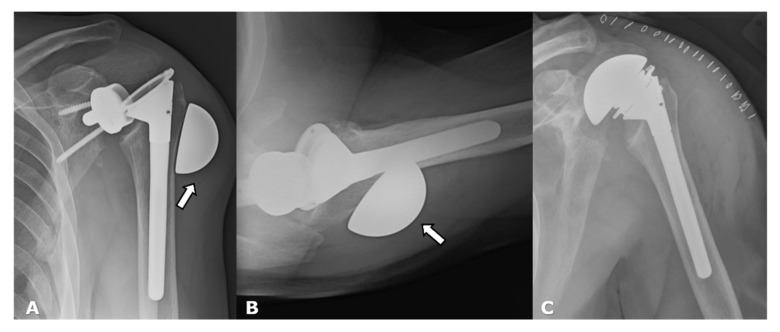
(**A**) Frontal and (**B**) axillary radiographs in a patient with RTSA shows dissociation of the glenosphere (arrows) from the metaglene, with displacement of the glenosphere into the posterior soft tissues. (**C**). The patient was treated via conversion to salvage HA.

**Figure 27:**
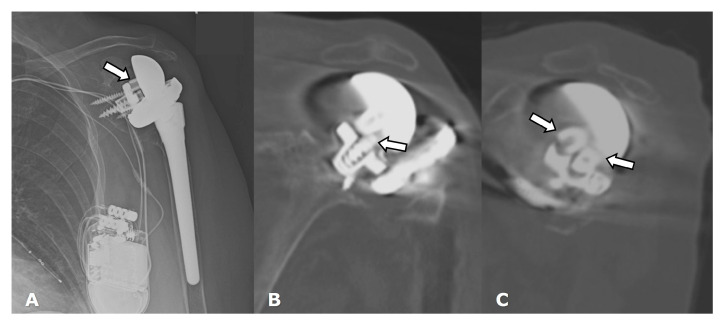
(**A**) Frontal radiograph and (**B**) coronal and (**C**) parasagittal CT images in a patient with RTSA show dissociation of the glenosphere from the metaglene/baseplate central screw (arrows).

**Figure 28:**
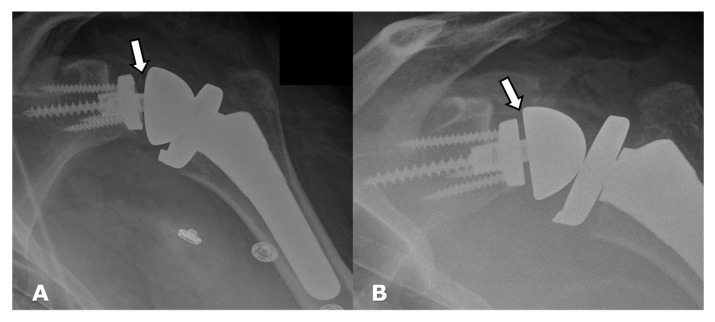
Frontal radiographs. (**A**). Immediate post-operative image following RTSA shows incomplete seating of the Morse taper into the baseplate, with resulting asymmetric alignment of the glenosphere (arrow). (**B**). Patient returned to the operating room for revision of the hardware, with post-operative image showing improved positioning of the Morse taper into the baseplate and improved glenosphere alignment.

**Figure 29:**
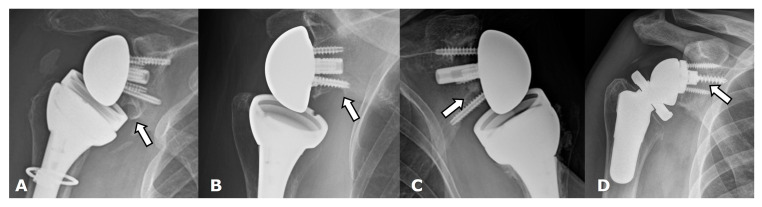
Frontal radiographs. Scapular notching grading by using Sirveaux Classification in four different patients with a reverse total shoulder arthroplasty. (**A**). Grade 1. (**B**). Grade 2. (**C**). Grade 3. (**D**). Grade 4.

Imaging—including radiography, ultrasonography (US), computed tomography (CT), magnetic resonance imaging (MRI), and, occasionally, nuclear medicine imaging—has a significant role in the initial pre-operative evaluation for shoulder arthroplasty, as well as in post-surgical follow up (Figure 4, Figure 5, Figure 6, Figure 7, Figure 8, Figure 9, Figure 10, Figure 11, Figure 12, Figure 13, Figure 14, Figure 15, Figure 16, Figure 17, Figure 18, Figure 19, Figure 20, Figure 21, Figure 22, Figure 23, Figure 24, Figure 25, Figure 26, Figure 27, Figure 28 and Figure 29). Imaging is critical in guiding the surgeon to select the appropriate arthroplasty type and surgical interventions to optimize patient outcomes. This review paper aims to discuss important pre-operative imaging considerations, including the rotator cuff evaluation, the glenoid morphology, and the glenoid version, as well as to review post-operative imaging of the various types of shoulder arthroplasties and to include normal post-operative appearances as well as imaging findings of complications. Imaging modalities discussed include radiography, ultrasonography (US), conventional computed tomography (CT), magnetic resonance imaging (MRI), and nuclear medicine imaging.

## 2. Clinical

### 2.1. Anatomy and Biomechanics

The ball-and-socket glenohumeral joint provides mobility at the expense of stability. The large humeral head and smaller glenoid articular surface create an unconstrained joint which is inherently unstable but provides a wide range of motion to include flexion, extension, abduction, adduction, circumduction, and rotation [2,3,4]. It consists of static (labroligamentous) and dynamic (muscular and tendinous) stabilizers. In the native shoulder, the rotator cuff provides inferior and medial force to the humeral head, producing compression of the convex humeral head within the concave glenoid fossa [2]. The deltoid exerts a superior force to abduct and elevate the humerus [2]. The force couples of the rotator cuff muscles and deltoid are balanced [5]. With large and massive rotator cuff tears, there is disruption of the force couple balance, with loss of the fulcrum, eventually leading to superior migration of the humerus and pseudo-paralysis [5].

### 2.2. Arthroplasty Indications

#### 2.2.1. Degenerative

Primary glenohumeral joint OA from age and activity-related cartilage degradation is the major indication for shoulder arthroplasty [6]. Asymmetric posterior glenoid wear and eventual biconcavity of the glenoid is the hallmark wear pattern in OA, due to posterior static subluxation. Cuff tear arthritis (CTA) is a variant of glenohumeral joint OA that results from a massive rotator cuff tear and unopposed deltoid muscle action, which results in superior migration of the humeral head (Figure 4 and Figure 5). Superior migration of the humeral head results in abnormal contact of the glenohumeral joint with the acromion. This abnormal contact leads to acetabularization of the undersurface of the acromion and femoralization of the humeral head and, eventually, severe glenohumeral joint osteoarthritis (Figure 4) [2,4,7]. Pseudo-paralysis can occur when there is superior translation of the humeral head, resulting in a loss of deltoid tension and an eventual inability to raise the arm above the horizontal plane.

#### 2.2.2. Trauma

The proximal humerus is a common fracture location in older patients; many of these fractures have minimal displacement and can be treated non-operatively with excellent clinical outcomes. However, approximately one-third of these fractures are treated surgically [8]. Elderly patients with displaced Neer three- and four-part fractures can have poor functional outcomes following non-operative treatment [2,9]. A recent randomized controlled trial compared RTSA with open reduction and internal fixation for complex proximal humeral fractures in elderly patients and showed that at two-year follow-up, there was a higher Constant score for RTSA comparted to the Constant score for reduction and internal fixation (ORIF) in complex fractures [8]. A randomized controlled trial by Johnson et al. showed that RTSA provided better shoulder function and patient satisfaction than in similar patients treated with HA, with a similar complication rate occurring with both surgical interventions [9]. Laas et al. compared the outcomes between HA and RTSA in elderly patients with dislocated three- and four-part humeral fractures and also showed better anterior elevation and a higher Constant score with RTSA in this patient population [10]. While RTSA has shown more predictable results than ORIF for proximal humeral fractures in the geriatric population, the outcomes of RTSA versus non-surgical treatment of three- and four-part fractures remain controversial.

#### 2.2.3. Inflammatory Arthritis

In inflammatory arthritis, such as rheumatoid arthritis (RA), there is cartilage destruction, with uniform joint space narrowing and concentric glenoid wear, resulting in a painful arthropathy. This wear pattern often results in severe medialization of the joint line. Tendon integrity can also be affected by the underlying inflammatory arthritis. End stage disease, which is frequently associated with superimposed OA, is a common indication for shoulder arthroplasty, commonly RTSA [11].

A systematic review by Cho et al. showed that RTSA performed in patients with RA provided pain relief and improvements in shoulder motion with higher Constant scores [11]. A retrospective review by Garcia et al. showed that ATSA performed in patients with inflammatory arthritis had improved clinical outcomes compared to RTSA but also higher rates of early revision [12].

#### 2.2.4. Instability

Glenohumeral joint instability, with resulting subluxations and dislocations and secondary post-instability arthritis, can be difficult to treat surgically, frequently due to young patient age as well as potential associated bone and soft tissue injuries [13]. A review by Cerciello et al. showed that complication and revision rates are more common following arthroplasties for glenohumeral joint instability when compared to arthroplasties performed for primary osteoarthritis, with ATSA associated with a lower revision rate than HA [13]. Higher complication and reoperation rates were observed in patients undergoing shoulder arthroplasty for instability following prior stabilization procedures compared to complication and reoperation rates in patients undergoing shoulder arthroplasty for primary osteoarthritis, likely due to increased surgical complexity, such as scar tissue from prior surgical intervention [13]. Clinical outcomes showed an increase in motion, function, and pain relief [13].

#### 2.2.5. Other

Other indications for shoulder arthroplasty include proximal humeral fracture sequelae such as post-traumatic osteoarthritis or malunion, humeral head osteonecrosis, and joint reconstruction following tumor resection.

## 3. Preoperative Imaging

Radiographs are the initial imaging study for patients with shoulder pain and frequently consist of the anterior–posterior (AP) view in external rotation, the AP view in internal rotation, and Grashey, axillary, and scapular Y views. Radiographs can aid in diagnosing arthritis and fractures and provide information on the status of the rotator cuff [14]. Frontal radiographs can be used to evaluate for glenoid erosion, humeral bone stock, and rotator cuff insufficiency [15]. The scapular Y view is used to evaluate acromial morphology. The axillary view can be used to assess anterior–posterior humeral head alignment and estimate glenoid version [15]. A narrowed acromiohumeral distance (<7 mm) with superior subluxation of the humeral head on frontal radiographs is consistent with a full-thickness rotator cuff tear (Figure 4). Greater tuberosity sclerosis and irregularity can indicate underlying rotator cuff arthritis. Acetabularization of the acromion and femoralization of the humeral head on radiographs suggest CTA (Figure 4) [16]. Several classification schemes exist for glenohumeral osteoarthritis, such as Samilson–Prieto, Kellgren and Lawrence, Weinstein, and Guyette, but are not widely used preoperatively, as they rely predominantly on the extent of osteophytes and joint narrowing and may not completely characterize the extent of glenoid arthritic change [17,18]. CT evaluation of the glenohumeral joint has been shown to better characterize glenoid morphology and static posterior humeral head subluxation [18]. Furthermore, newer MRI techniques such as zero echo time (ZTE) imaging can be used to create CT-like high-contrast bone images and 3D volume-rendered images which can be useful for preoperative evaluation [19].

Preoperative imaging of the osseous and soft tissue structures helps determine the type of arthroplasty that will be used. Preoperative imaging evaluation may also include CT, MRI, and US in addition to initial radiography.

CT is used to evaluate the glenoid morphology, bone stock, version, and inclination, which may be affected by arthritis, trauma, prior hardware, tumor resection, and congenital dysplasia [20,21]. On CT, the shape of the axillary margin of the scapula is assessed for glenoid component positioning [22]. The glenoid often undergoes erosive changes secondary to altered mechanics and arthritis, resulting in altered morphology, which can be described by using the modified Walch classification (Table 1) (Figure 6) [2,3,6,14,16,23,24,25,26,27].

Osseous erosion of the glenoid results in reduced bone stock available to support the glenoid component [6]. Adequate glenoid bone stock is necessary to allow for correction of the glenoid version if needed, as well as for placement of the glenoid component hardware [23]. CT can assess glenoid bone stock, which should be at least 2 cm in depth centrally (Figure 7) [22,23].

Glenoid version is measured by drawing a line from the superior medial scapular border to the center of the glenoid on the axial image at or just inferior to the tip of the coracoid. A line is drawn perpendicular to this line, and the glenoid version angle is measured between the perpendicular line and a line connecting the anterior and posterior margins of the glenoid articular surface [3,14,23,26,28]. Other three-dimensional methods of estimating bone loss exist, including evaluation of the glenoid vault shape [29]. Asymmetric posterior glenoid bone loss leads to altered glenoid version; CT is the preferred imaging modality to evaluate for glenoid version (Figure 8) [7]. In native shoulders, the glenoid has between 2° of anteversion and −9° of retroversion [6,23,30]. Abnormal version must be corrected to prevent arthroplasty failure from asymmetric loading forces. Asymmetric reaming can be used to correct abnormal glenoid version but can result in the removal of a large amount of anterior bone [25]. Newer approaches utilizing minimal reaming without glenoid retroversion correction have shown promise [31]. Other alternatives to eccentric reaming include augmented glenoid components or placement of an RTSA rather than an ATSA. Bone grafting can also be used to correct glenoid version, with failure of graft incorporation and graft resorption and hardware failure potential complications. Failure of graft incorporation and graft resorption can occur in up to 60% of cases. Subsequently, metal augmentation of osseous defects has fallen into favor [32].

The glenoid inclination angle is used to characterize glenoid bone loss in the coronal plane, to evaluate for superior bone loss, particularly in patients who are being evaluated preoperatively for RTSA, as significant superior bone loss can cause abnormal angulation of the base plate (Figure 9) [6]. Glenoid inclination varies but should be between 0 and 10 degrees [33].

Axial imaging of the distal humerus can be used to assess the humeral torsion angle and may be helpful in future studies evaluating implant stability and post-operative motion.

Three-dimensional (3D) CT preoperative planning software can be used to aid in positioning and fixation of the glenoid component (Figure 10) [14,28]. Patient-specific instrumentation (PSI) has been used to improve accuracy in restoring glenoid version. With PSI, preoperative 3D CT images are used to create a custom-made drilling guide that is used intraoperatively to improve positioning and orientation of the glenoid component, particularly of the baseplate and screws in RTSA [21,28,34]. A systematic review by Lilley et al. in 2022 showed a high level of accuracy in glenoid component placement when using preoperative 3D planning software for RTSA [35].

Humeral bone stock is also an important preoperative consideration, particularly in primary arthroplasties performed for trauma, prior infection, or tumor, as well as for revision arthroplasties, and both the quantity as well as the quality of the bone should be reported [36]. Radiographs are often adequate for assessing humeral bone loss preoperatively.

Osteophytes should be noted on preoperative imaging to help guide an operative approach to optimizing exposure and hardware placement [6]. Additionally, subchondral cyst-like changes should be noted, as they may alter surgical planning [6]. The acromion should also be assessed for morphological changes from prior surgery or rotator cuff pathology, as excessive thinning of the acromion can predispose to fracture in RTSA. Pre-operative visualization of os acromiale may help in post-operative discernment of acromial fractures versus normal anatomic variation.

MRI and US are the imaging modalities of choice for evaluation of the rotator cuff tendons, while CT and MRI are useful for evaluating the shoulder muscle bulk. US is less accurate in the assessment of rotator cuff muscular atrophy, particularly of the subscapularis, which can limit its use in the preoperative setting. An intact or reparable cuff is essential for an ATSA, while an intact deltoid is needed for an RTSA. Muscle atrophy should be evaluated. Greater than 50% fatty infiltration of the rotator cuff musculature on MRI is suggestive of an irreparable cuff [16]. Care must be taken to assess muscle bulk and quality medially adequately to ensure muscle retraction is not mimicking atrophy.

Deltoid dehiscence and fatty infiltration can be identified on MRI [16]. MRI and US are also excellent in the evaluation of the coracoacromial ligament [16].

## 4. Post-Operative Imaging and Imaging of Complications

### 4.1. Imaging Techniques

ACR Appropriateness Criteria^®^ Imaging After Shoulder Arthroplasty recommends radiographs for routine follow-up of all patients with a primary shoulder arthroplasty, as well as the initial imaging for symptomatic patients with a primary shoulder arthroplasty (Figure 11) [37]. Shoulder arthroplasty has a complication rate of up to 40% and a revision rate of up to 11% [37,38]. Post-operatively, symptoms of pain, apprehension, and decreased range of motion should raise suspicion for complication.

If infection is suspected, image-guided shoulder aspiration is recommended, and it is thought that US, MRI, and nuclear medicine three-phase bone scan/white blood scan and sulfur colloid scan “may be appropriate” (Figure 12) [37]. Once infection has been excluded, MRI or CT are the preferred modalities to evaluate for loosening; US, MRI, or CT arthrography can be used to evaluate for a rotator cuff tear [37].

Radiographs are the most useful tools in evaluating for glenoid erosion, loosening, fractures, and heterotopic ossification (Figure 13, Figure 14, Figure 15, Figure 16, Figure 17, Figure 18, Figure 19C and Figure 20). Radiographs can be used to assess the adjacent bones and soft tissues as well as to assess the implant positioning and integrity (Figure 16, Figure 21A, Figure 22, Figure 23, Figure 24, Figure 25 and Figure 26).

US has several advantages, including widespread availability, relatively low cost, dynamic capabilities, high resolution, and a lack of ionizing radiation. This imaging modality is, however, very operator-dependent. US is highly useful in the evaluation of the rotator cuff integrity following shoulder arthroplasty and lacks the metallic artifact that occurs on MRI and CT (Figure 21B). US can also evaluate bursal fluid, joint effusion, synovitis, and osseous irregularity [39].

CT is readily available and relatively quick to perform but uses ionizing radiation. Although artifacts from hardware occur with CT, newer metal reduction protocols for CT have decreased the occurrence of artifacts. Dual-energy CT (DECT) can also be used to decrease metal artifact and improve overall image quality [40,41]. CT is useful in detecting loosening, fractures, heterotopic ossification, hardware integrity, rotator cuff integrity, fatty infiltration of rotator cuff, and deltoid musculature and fluid collections (Figure 19A,B and Figure 27). CT can also be used to evaluate tendon integrity, but evaluation can be limited in the setting of non-displaced and partial-thickness tears.

MRI provides superior soft-tissue contrast and multiplanar capabilities, without the use of ionizing radiation. However, MRI is more expensive, less readily available, and time consuming and can be limited by implanted medical devices or other retained foreign bodies, as well as by claustrophobia. The use of MRI in the evaluation of arthroplasties has previously been limited due to susceptibility artifacts created by the metallic implants. However, artifacts can be decreased by using lower-field-strength magnets (1.5 T rather than 3 T), using fast-spin echo sequences rather than gradient-recalled echo sequences, aligning the implant parallel to the external magnetic field, and placing the implant close to the isocenter [28,42]. The use of short tau time inversion recovery imaging and chemical shift water–fat separation sequences should be used rather than traditional fat saturation methods [28,42]. Decreasing voxel size by decreasing the slice thickness and/or by increasing the frequency acquisition matrix and increasing bandwidth also reduces the occurrence of artifacts [28,42]. Newer techniques such as slice encoding for metal artifact correction (SEMAC) and multiple-acquisition variable-resonance combination (MAVRIC) are helpful in decreasing artifact [28,42,43,44]. MRI is useful in detecting loosening, fractures, rotator cuff integrity, and fluid collections (Figure 12D).

Technetium-99 m three-phase bone scan imaging is sensitive for arthroplasty failure but is limited in determining the etiology [37,45].

### 4.2. General Complications

#### 4.2.1. Loosening and Hardware Dissociation

Radiographically, aseptic loosening is evident as lucency surrounding the implant or a change in implant positioning (Figure 14, Figure 15 and Figure 16) [46]. Loosening will appear as progressive radiolucency or radiolucency greater than 2 mm surrounding the hardware. Component subsidence as well as pedestal formation (endosteal new bone formation below the distal end of the humeral stem that usually extends over 50% of the canal) can be seen in addition to radiolucent line formation in humeral component loosening [36]. Subsidence of the humeral component is suggested when the humeral head component is greater than 5 mm below the greater tuberosity [23]. In all arthroplasties, the humeral stem should be centered within the humeral diaphysis.

MRI can identify areas of osteolysis around hardware components in aseptic loosening, often of lobulated, intermediate signal intensity [23,42]. Loosening manifests as a thin linear gap of signal abnormality along the bone–hardware, bone–cement, or cement–hardware junction. Synovitis is frequently present, due to polyethylene liner wear, and can be seen as fluid with intermediate signal intensity debris, often with a frond-like appearance [42]. CT is also highly sensitive for the detection of osteolysis occurring with aseptic loosening.

Hardware can fracture or pull away from the bone. Component disengagement is also possible. In the setting of RTSA, glenosphere dissociation, in which the glenosphere separates from the metaglene/baseplate, has an incidence of approximately 3.2% [28]. Radiographs and CT can be helpful in evaluating hardware integrity and detecting complications such as hardware fracture or component dissociation (Figure 16, Figure 22, Figure 23, Figure 24, Figure 25, Figure 26 and Figure 27). Additionally, hardware can be improperly or sub-optimally positioned intraoperatively (Figure 28), emphasizing the importance of obtaining immediate post-operative radiographs.

#### 4.2.2. Stress Shielding and Fractures

Stress shielding occurs in 9% of ATSA and HA and can lead to aseptic loosening and periprosthetic fracture [2,26,28]. It results from altered biomechanics and stress distribution caused by the humeral component, which results in osseous resorption around the proximal humeral stem [23,28]. On radiographs, stress shielding is manifested as cortical thinning and cortical tunnelling [23].

Peri-prosthetic humeral fractures may occur intraoperatively during implant placement or post-operatively. Intraoperative fracture occurs more commonly in revision shoulder arthroplasty than in primary arthroplasty, particularly when removing the humeral component during revision surgery [2,28]. Fractures involving the proximal stem tend to be more stable and are treated conservatively, while more distal fractures are often treated operatively, either via revision stem placement or open reduction internal fixation [47].

Immediate post-operative radiographs should be obtained to evaluate for dislocation and intraoperative fracture (Figure 17). Radiographs should also be obtained following trauma.

MRI with metal artifact reduction can help identify periprosthetic fractures, either by directly depicting a fracture line or by demonstrating bone marrow edema, which can be seen with acute and early subacute fractures. MRI can also identify screw malpositioning that can result in the impingement of neurovascular structures or other adjacent soft tissues. CT is also useful in detecting periprosthetic fractures.

#### 4.2.3. Periprosthetic Joint Infection (PJI)

PJI of the shoulder has been reported in 1.1–4% of ATSA and in 3.8–18% of RTSAs [39,48,49], which may be complicated by the fact that RTSA is often used in revision surgeries, which are at a higher risk of PJI. The risk of PJI is three times higher in arthroplasties following trauma compared to elective arthroplasties and has been found to be 2.5 times more common in males compared to females [39,49]. Other risk factors include young age, smoking, and hemodialysis [7].

Staphylococcus is a common pathogen in shoulder PJI. *Cutibacterium acnes* (*C. acnes*), formerly *Propionibacterium acnes*, is the other major organism which may be detected with shoulder arthroplasty infections (31–70% of PJI of the shoulder) and can occur up to two years post-operatively [48,49,50]. *C. acnes* is a non-spore-forming, anaerobic, Gram-positive bacillus commonly found in hair follicles, sebaceous glands deep in the dermis, conjunctiva, respiratory tract, gastrointestinal tract, and external auditory canal [7,39,49,50]. It often resides in a biofilm, where it is isolated from the patient’s immune system, avoiding phagocytosis [49,50]. Patients often present with pain and stiffness, without fever, and with normal laboratory values, including white blood cell count, erythrocyte sedimentation rate, and C-reactive protein [39,49,50]. Due to the slow rate of growth and often low concentration of the bacteria within the biofilm, cultures must be kept for a minimum of 14 days. Positive cultures in the absence of clinical symptoms are not thought to indicate infection; 24–50% of shoulders with revision arthroplasties have been shown to be culture-positive, but true infection is suspected in only 5–25% of these cases [51]. Currently, joint fluid is often evaluated for the presence of α-defensin, an antimicrobial peptide released by neutrophils, which is highly sensitive and specific for PJI (85–100%) [39]. A variety of treatment options exists for PJI, including one-stage, two-stage, or three-stage revisions [47].

Infected arthroplasties may appear radiographically normal. Osteolysis and periostitis may also be present (Figure 12A,B), and the imaging findings may be difficult to distinguish from aseptic loosening. Humeral stem loosening is considered pathognomonic for prosthetic joint infection [52].

In infection, MRI usually shows inflammatory synovitis with a hyperintense, thickened, and lamellated appearance of synovium [42]. A fluid collection/rim enhancing abscess and sinus tract may be present (Figure 12C).

US can be used to guide aspiration in patients with suspected PJI and to evaluate for soft tissue fluid collections in cases of seromas, hematomas, and abscesses (Figure 12D). Importantly, US helps the radiologist avoid passing through potentially infected tissues, such as wounds, ulcers, cellulitis, and subcutaneous fluid collections when aspirating the joint [39].

An isolated, indium-labeled white blood cell (WBC) study has proven to be poor at detecting periprosthetic joint infection (PJI) caused by low-virulence organisms [22].

#### 4.2.4. Rotator Cuff Tendon Tears

Subscapularis tendon tears are a common complication following shoulder arthroplasty [2,14]. Intraoperatively, subscapularis tenotomy, subscapularis peel, or lesser tuberosity osteotomy are often performed to access the glenohumeral joint via a deltopectoral approach, making the subscapularis tendon susceptible to post-operative tearing [2,14,39]. Inadequate healing of the subscapularis tendon as well as subscapularis tears can result in pain, anterior instability, loss of function, and in some cases, loosening of the glenoid component due to abnormal forces; thus, proper repair of the subscapularis tendon is essential to arthroplasty longevity and avoidance of revision [5,26,39,53,54]. A subscapularis-sparing surgical approach through the rotator interval may help reduce post-operative subscapularis failure in certain patient populations. However, there is decreased glenohumeral joint exposure using this approach, which can result in residual osteophytes, non-anatomic humeral neck osteotomies, and improper sizing of the humeral implant [53,54]. A review by Lee et al. in 2021 showed that a subscapularis-sparing approach resulted in decreased pain and an improved active range of motion, with low complication rates, up to two years post-operatively [54]. Due to the difficulty of exposure, this approach may be best used in limited cases with minimal osteophyte burden and little to no glenoid deformity.

In the case of subscapularis tendon failure due to surgical approach, radiographs can identify fractures through the lesser tuberosity osteotomy [14]. If the tendon avulses from the bone due to complications from a subscapularis tenotomy or peel, there may be subtle anterior translation of the humeral component compared to the glenoid [14].

Supraspinatus and infraspinatus tendon tears result in superior migration of the humerus with eventual anterosuperior instability. Superior migration of the humeral head on frontal radiographs and anterior migration of the humeral component on axillary radiographs suggest rotator cuff insufficiency; US is useful in identifying tears (Figure 21) [14,23].

### 4.3. Unique Complications

Complications unique to RTSA, including scapular notching, scapular fractures, and acromial fractures are discussed in detail in the section on RTSA.

## 5. Arthroplasty Types

### 5.1. Anatomic Arthroplasties

HAs, including resurfacing arthroplasties, replace only the humeral side of the joint, while total arthroplasties replace both the humeral and glenoid articular surfaces. Anatomic arthroplasties maintain the normal orientation of the ball-and-socket anatomy of the shoulder and require an intact rotator cuff for optimal function. Depending on the length of the stem, they can be classified as resurfacing, stemless, short-stem, or stemmed prostheses.

#### 5.1.1. Humeral Head Resurfacing/Stemless Hemiarthroplasty (HHRA)

With HHRA, the humeral head articular surface is replaced with a metallic hardware cap. With stemless HA, a humeral head osteotomy is performed, and the humeral head is replaced with a metallic component containing a short metaphyseal fixation component [23]. These arthroplasties require an intact or repairable rotator cuff, as well as intact glenoid cartilage. HHRA and stemless shoulder arthroplasty are used to treat isolated humeral head abnormalities, such as osteonecrosis, fractures, humeral head defects related to prior instability, and humeral cartilage loss, particularly in younger, active patients [3,23,28,37,55,56]. Resurfacing arthroplasties are occasionally used to replace a part of the humeral head surface such as for the treatment of large Hill–Sachs or reverse Hill–Sachs lesions [23,55]. Occasionally, HHRA can be paired with an inlay component, creating a total anatomic type arthroplasty, without humeral head osteotomy, preserving proximal anatomy and bone stock (Figure 1) [55]. These types of arthroplasties permit preservation of both glenoid and humeral bone stock, allowing for increased ease of future arthroplasty revisions [2]. HHRA also avoids difficulties encountered during stem removal when revision surgery is required. These arthroplasties decrease operative time and have a lower risk of periprosthetic fracture [2,55].

Glenoid component loosening is more common than complications involving the humeral stem in ATSA [57,58], and this is avoided with HHRA. Importantly, HHRA allows for a more anatomic restoration of the native glenohumeral geometry and better replicates glenohumeral biomechanics than HA, thus decreasing the rate of glenoid implant complications and longevity concerns [55,56]. Fractures, bone loss or fracture malunion may prevent appropriate positioning of the humeral component [57].

#### 5.1.2. Hemiarthroplasty (HA)

HA consists of a spherical metallic articular surface humeral head component attached to a humeral stem. No glenoid component is present, and the humeral head component articulates with the native glenoid [6,16,28]. These arthroplasties require an intact or repairable rotator cuff, with adequate humeral bone stock. HA is used to treat isolated humeral pathologies, such as osteonecrosis and humeral fractures. HA can also be used as an alternative to total arthroplasties when there is inadequate glenoid bone stock [23]. An intact coracoacromial arch is important to support the arthroplasty and prevent humeral head anterosuperior escape [14]. With severe OA, there is decreased pain relief and function and higher revision rates with HA compared to ATSA [2,26].

In a younger patient with rotator cuff deficiency but an intact glenoid, HA can be used if there is an intact coracoacromial arch and other criteria are met, to allow increased function until an RTSA is needed [7]. Care should be taken to distinguish an HA performed in this scenario from a typical HA with subsequent rotator cuff failure.

A common complication of HHRA/HA is native glenoid erosion/wear, manifesting as progressive asymmetric joint space narrowing, which may necessitate revision to ATSA (Figure 13) [2].

#### 5.1.3. Anatomic Total Shoulder Arthroplasty (ATSA)

ATSA is the most common arthroplasty that is used for OA [6]. An intact or repairable rotator cuff as well as adequate glenoid and humeral bone stock is necessary for a functional ATSA [2,16]. The humeral component is a minimally constrained implant with either a cemented or pressed fit stem, a metallic spherical head, and an adjustable neck angle (Figure 2) [16,26,28]. The glenoid component is most often a radiolucent polyethylene implant fixed to the underlying bone, often via either a central keel or central pegs, which are secured with polymethylmethacrylate cement [23,26,28]. Various other designs of glenoid components are available, such as hybrid uncemented components with press-fit pegs or metallic pegs, e uncemented metal-backed components, and augmented components.

The most frequent complication in ATSA is loosening of the glenoid component, which is reportedly seen in up to 37% of cases (Figure 14) [6,28,31,59]. Metal back-to-glenoid prostheses have been shown to have a higher failure rate [31]. Cemented all-polyethylene glenoid components have been shown to have high survivorship [21,31]. Currently, hybrid glenoid components with posts are routinely used to attempt to incorporate biology and improve implant longevity. On radiographs, the glenoid component should be well aligned along the glenoid. Although the polyethylene component is radiolucent, the keel or pegs contain linear radiopaque markers [23]. Lucency less than 1.5 mm in thickness around the keel or pegs is unlikely to represent loosening [23]. Rotator cuff failure (9%) and humeral loosening (1%) are less common complications [31].

If a cuff tear occurs in an ATSA, the humeral head is no longer centered on the glenoid, resulting in increased forces along the glenoid component and polyethylene wear. The resulting repetitive eccentric loading of the glenoid with humeral head movement produces a rocking-horse mechanism of glenoid component loosening [5,55]. Dislocation following ATSA is often secondary to rotator cuff deficiency, coracoacromial arch insufficiency, humeral component malrotation, glenoid dysfunction, and capsular failure [16].

### 5.2. Reverse Arthroplasty

#### Reverse Total Shoulder Arthroplasty (RTSA)

Reverse arthroplasties reverse the ball-and-socket anatomy, with the humerus becoming the socket and the glenoid functioning as the ball. Approved for clinical use in the United States in 2004, the RTSA is now the most common operative treatment for glenohumeral joint osteoarthritis secondary to rotator cuff arthritis and is increasingly used for primary shoulder arthroplasties [7]. These implants require adequate humeral and glenoid bone stock as well as a functional deltoid muscle.

The RTSA prosthesis consists of a humeral component, a polyethylene insert, a glenosphere, and a metaglene/baseplate (Figure 3). The baseplate is secured to the glenoid by screws, including a long central screw [6]. The glenosphere is attached to the baseplate with a more taper, or in older implants, a central screw [22,23].

The RTSA reverses the normal anatomy of the shoulder joint, replacing the glenoid component with a ball and replacing the humeral head with a socket. The reverse anatomy repositions the center of rotation and utilizes distalization of the humerus, thus restoring the tension of the deltoid muscle, which becomes the primary muscular force to move the shoulder [4,45]. The RTSA is a semi-constrained design which provides a stable fulcrum, as well as static stability and increased range of motion [45,60]. This prosthesis restores active arm elevation, but the constrained nature of the arthroplasty limits internal and external rotation [16,28].

Initially used to treat patients with irreparable rotator cuff tears with secondary arthropathy, pain, and pseudo paralysis, RTSA produced excellent clinical outcomes, with respect to increased range of motion and decreased pain but had high rates of complications, including instability, periprosthetic fractures, infection, scapular notching, dislocation, hematoma, hardware loosening, and hardware dissociation [7,22]. Newer designs of the RTSA have decreased complication rates and are allowed for greater use.

Current indications for RTSA include irreparable rotator cuff with painful arthropathy, massive irreparable cuff tears without significant OA in older patients, advanced glenohumeral joint OA with an intact rotator cuff, pseudo-paralysis, fractures, previous septic arthritis, inflammatory arthritis, osteonecrosis, tumor-related reconstruction, and revision surgeries for failed HA and ATSA [2,4,6,16,22,23,61,62]. RTSA has been shown to provide good clinical outcomes regardless of the preoperative diagnosis. A systematic review by Coscia showed that the best outcomes are seen in primary glenohumeral joint OA and massive rotator cuff tear with or without glenohumeral joint OA, with less consistent outcomes seen with trauma or revision arthroplasty [61].

RTSA can also be used to treat glenohumeral joint OA in the presence of an intact rotator cuff [63] and can provide good clinical and functional outcomes in these patients. Altered glenoid morphology in OA can cause poor outcomes in ATSA, and poor glenoid morphology can be mitigated with the use of RTSA, as the glenoid component position does not affect stability as much as anatomic glenoid components [63]. Glenoid bone loss and abnormal glenoid version are more easily corrected with reverse arthroplasty hardware [63].

Patients with RA often have both rotator cuff tears and advanced glenohumeral arthropathy as a sequalae of their disease. A systematic review by Cho et al. showed that RTSA performed in patients with RA showed decreased pain but also significant improvement in functional shoulder motion [11], with an overall complication rate of 20.4% and an overall revision rate of 7.3% [11].

RTSA has been used to treat complex proximal humeral fractures, and its use for this indication has increased by over 400% between 2005 and 2012 [60]. Proximal humeral fractures are the second most common upper extremity fracture and account for 5% of all adult fractures [64]. As these fractures frequently occur in elderly patients with complex fracture patterns and osteoporosis, treatment can be difficult and was previously limited to conservative management or HA, often resulting in good pain relief but unpredictable functional outcomes [64]. A prospective randomized control study by Lopiz et al. compared non-operative management versus RTSA in the treatment of displaced Neer three- and four-part proximal humerus fractures in geriatric populations (age 80 years or older) showed improvement in pain, but not functional status in the RTSA group [64]. A recent systematic review by Paras showed that, when compared to patients who had elective RTSA, patients who had RTSA performed for fracture had significantly lower forward elevation, abduction, and external rotation but no difference in other complications such as loosening, revision, nerve injury, post-operative stiffness, infection, and dislocation [60].

On routine post-operative follow up radiographs of RTSA, the glenosphere should align with the humeral component, separated by the radiolucent polyethylene insert. The metaglene/baseplate should be flush with the glenoid. The superior, anterior, and posterior baseplate screws are bicortical, and the inferior screw should be within the scapula [4,22]. The space between the glenosphere and the humeral component will vary depending on the size of the radiolucent polyethylene spacer, but the glenosphere should align with the humeral cup. The humeral component should be centered in the diaphysis.

Arthroplasty positioning should also be assessed to evaluate for instability and/or dislocation. RTSA dislocation most commonly occurs in the first 6 weeks post-operatively. often when the patient reaches behind their back or pulls up their pants. The anterior lip of the humeral tray levers against the glenoid and dislocates. Immediate post-operative radiographs should be obtained to ensure proper alignment and component placement [4,22,28]. Anterior-superior “escape” is common with RTSA and occurs when the humeral component dislocates in the anterior–superior direction secondary to a deficient rotator cuff and unopposed pull of the deltoid muscle (Figure 22) [2,4,22,23,26].

The periarticular soft tissues should be evaluated for the development of heterotopic ossification (Figure 20).

Acromial and scapular spine fractures have an incidence of at least 5% and may be higher due to under-diagnosis following RTSA [2,4,7,26,65,66]. These fractures are usually considered fatigue-type stress injuries and are caused by superior glenoid baseplate screw stress risers and deltoid over-tensioning [4,14,65,66]. Acromioclavicular joint osteoarthritis, loss of coracoacromial ligament function, and prosthesis design are also thought to be potential etiologies [66]. However, in a recent series of patients with this type of fracture following RTSA, most fractures were related to a fall, and the authors proposed that placement of an RTSA exposes the acromion and places it at increased risk for fracture due to direct trauma. In this series, non-traumatic fractures were associated with poor bone quality [66]. Both fractures can result in significant loss of shoulder function as compressive forces are weakened, as the deltoid anchor becomes compromised [47,66]. Treatment is usually conservative management, with operative intervention having varied results [47,65]. These fractures are best seen on axillary radiographs, CT, and MRI (Figure 18 and Figure 19).

Scapular notching, a unique complication of RTSA, involves erosive change in the inferolateral scapular margin adjacent the glenoid due to repetitive contact and mechanical impingement of the medial border of the humeral component with the inferior border of the scapula during arm adduction [2,4,7,14,16,22,23,26,28,67]. The inferior axillary margin of the scapula should be evaluated on post-operative imaging for notching and graded appropriately by using the Sirveaux classification (Figure 29) (Table 2) [68]. It is unclear if scapular notching influences clinical outcomes, although it may affect glenoid fixation and glenoid component stability and, ultimately, hardware integrity [7,14,26,28,67,69]. Scapular notching occurs more commonly in RTSA with medialized centers of rotation and is reported in up to 50–66% of patients with RTSA in the first 2 years following surgery [23]. It has also been shown that a larger glenosphere size can decrease scapular notching [67]; smaller glenospheres with eccentricity have been shown to have slightly increased rates of scapular notching compared to larger glenospheres, but both have similar clinical outcomes [69]. Lateralization of the center of rotation, inferior angulation of the glenoid component, and decreased humeral component neck angle can also influence scapular notching. Newer generations of RTSA have a lateralized center of rotation, which improves loading and reduces impingement [45,60]. This lateralization can be achieved by using bone autograft under the baseplate or with metal augmented baseplates, both of which have been shown to have similar 2-year clinical outcomes [70]. Some implants designs also incorporate over-lateralization in the glenosphere. A recent systematic review by Nunes et al. showed that lateralized RTSAs improve clinical and functional outcomes similar to medialized implants but may have a lower risk of scapular notching [45]. Another review by Burden et al. showed significant improvement in patient-reported outcome measurements and low complications in RTSA regardless of implant design [62]. While laterization may decrease scapular notching and improve biomechanics of remaining rotator cuff tendons/function, the increase torque forces at the bone–metal interface of the glenoid component remain a concern.

### 5.3. Revision Shoulder Arthroplasty

The increasing use of primary shoulder arthroplasty has also resulted in an increased incidence of revision shoulder arthroplasty. Arthroplasty survival rate is dependent upon the underlying primary condition, patient age, patient activity level, and type of hardware [58,71]. RTSA is often used in revision arthroplasty to treat failed HA and ATSA, in particular to treat glenoid component loosening in ATSA [15]. Outcomes for these revision arthroplasties are largely dependent on the underlying reason for revision, with revisions for hardware loosening or glenoid wear having better outcomes than those performed for infection or instability [46,58,72]. Although clinical outcomes have been shown to be poorer in revision arthroplasties compared to primary arthroplasties, post-operative pain and function are often improved in revision arthroplasties [36,46,58]. With a failed RTSA, if revision RTSA is not possible, conversion to HA can be performed [73].

## 6. Conclusions

Shoulder arthroplasty has become an important treatment option for a variety of conditions affecting the shoulder including advanced primary OA, post-traumatic and post-infectious arthritis, inflammatory arthritis, osteonecrosis, certain fractures, and rotator cuff disease. Several types of shoulder arthroplasties are currently available, each with specific indications and unique complications in addition to general hardware-related or surgery-related complications. The radiologist must understand the role of preoperative imaging, which can guide the orthopedic surgeon in selecting the appropriate arthroplasty type. Knowledge of their normal post-operative imaging findings and potential complications is essential to optimize long term patient outcomes.

## Figures and Tables

**Table 1:** Modified Walch Classification. Glenoid shape is classified by erosion location as well as humeral head position.

Type	Description
A1	Mild central glenoid erosion with the humeral head centrally positioned.
A2	Major central glenoid erosion with the humeral head centrally positioned; a line that connects the native anterior and posterior glenoid rims transects the humeral head.
B1	No glenoid erosion; posteriorly subluxed humeral head with posterior joint space narrowing and osteophytes.
B2	Biconcave glenoid due to posterior erosion and retroversion, with posteriorly subluxed humeral head.
B3	Monoconcave glenoid with significant posterior glenoid wear with retroversion of at least 15 degrees or subluxation of 70% or both.
C	Retroverted glenoid with glenoid dysplasia.
D	Anteverted glenoid or anteriorly subluxed humeral head.

**Table 2:** Sirveaux classification. Extent of scapular erosion present on radiographs is used to determine the grade of scapular notching.

Grade	Finding
1	Lucency extends to scapular pillar
2	Lucency contacts the inferior glenoid screw
3	Lucency extends over the inferior glenoid screw
4	Lucency extends under the metaglene/baseplate

## Data Availability

No new data were created or analyzed in this study. Data sharing is not applicable to this article.
